# Home-based deep sleep modulation in Parkinson’s disease: extension study on long-term feasibility and sleep-related outcomes

**DOI:** 10.1038/s41531-026-01461-1

**Published:** 2026-07-10

**Authors:** Jana Horlacher, Nora-Hjördis Moser, Simon J. Schreiner, Renske Sassenburg, Lydia Kämpf, Sophia D. Schibler, Valentino Mutschler, Esther Werth, Philipp Valko, Jelena Skorucak, Laura Ferster, Walter Karlen, Reto Huber, Christian R. Baumann, Angelina Maric

**Affiliations:** 1 Department of Neurology, University Hospital Zurich, University of Zurich, Zurich, Switzerland; 2 Center of Competence Sleep & Health Zurich, University of Zurich, Zurich, Switzerland; 3 Child Development Centre, University Children’s Hospital Zurich, University of Zurich, Zurich, Switzerland; 4 Tosoo AG, Zurich, Switzerland; 5 Institute of Biomedical Engineering, Ulm University, Ulm, Germany; 6 Department of Child and Adolescent Psychiatry, University Hospital of Psychiatry, University of Zurich, Zurich, Switzerland

**Keywords:** Diseases, Health care, Medical research, Neurology, Neuroscience

## Abstract

This study assessed feasibility and effects of a randomised two-week, at-home intervention with phase-targeted auditory stimulation (PTAS) of slow waves in Parkinson’s disease (PD). We reproduced previously reported enhancement of slow wave activity and confirmed indications of improved patient-reported outcomes. Together with the high adherence and data quality, this suggests that home-based PTAS protocols are a feasible method for investigating the long-term therapeutic potential of deep sleep enhancement in PD.

## Introduction

Sleep-wake disturbances are among the most prevalent non-motor symptoms in Parkinson’s disease (PD)^1,2^. In this context, disruption of deep sleep, also known as slow wave sleep (SWS), is frequently observed in PD^3,4^ and has been associated with excessive daytime sleepiness (EDS), impaired cognitive function, and decreased quality of life^1,5–8^. While pharmacological methods for SWS enhancement have shown some success in improving subjective and objective symptoms of EDS in PD patients, they are accompanied by mild to moderate side-effects and de novo obstructive sleep apnea and parasomnia^9,10^. These limitations together with the importance of SWS for physical restoration, overall health and daily functioning^11,12^ emphasise the need for alternative methods to enhance SWS that are both safe and effective.

In recent years, phase-targeted auditory stimulation (PTAS) has emerged as a promising non-pharmacological method to modulate SWS by presenting auditory stimuli at a specific phase of slow waves^13–17^, the predominant electroencephalographic (EEG) oscillation during SWS. A recent proof-of-concept study in PD demonstrated that at-home PTAS applied over three nights can increase slow wave activity (SWA), namely the spectral power in the slow wave frequency range, in addition to first indications that this can lead to a reduction of subjective daytime sleepiness over time^17^. However, it remains unclear whether PTAS can be feasibly and effectively self-administered over extended time periods in these patients. Additionally, it needs to be clarified if subjective improvements go along with tangible, objective symptom improvements of sleep-wake disturbances.

**Fig. 1:**
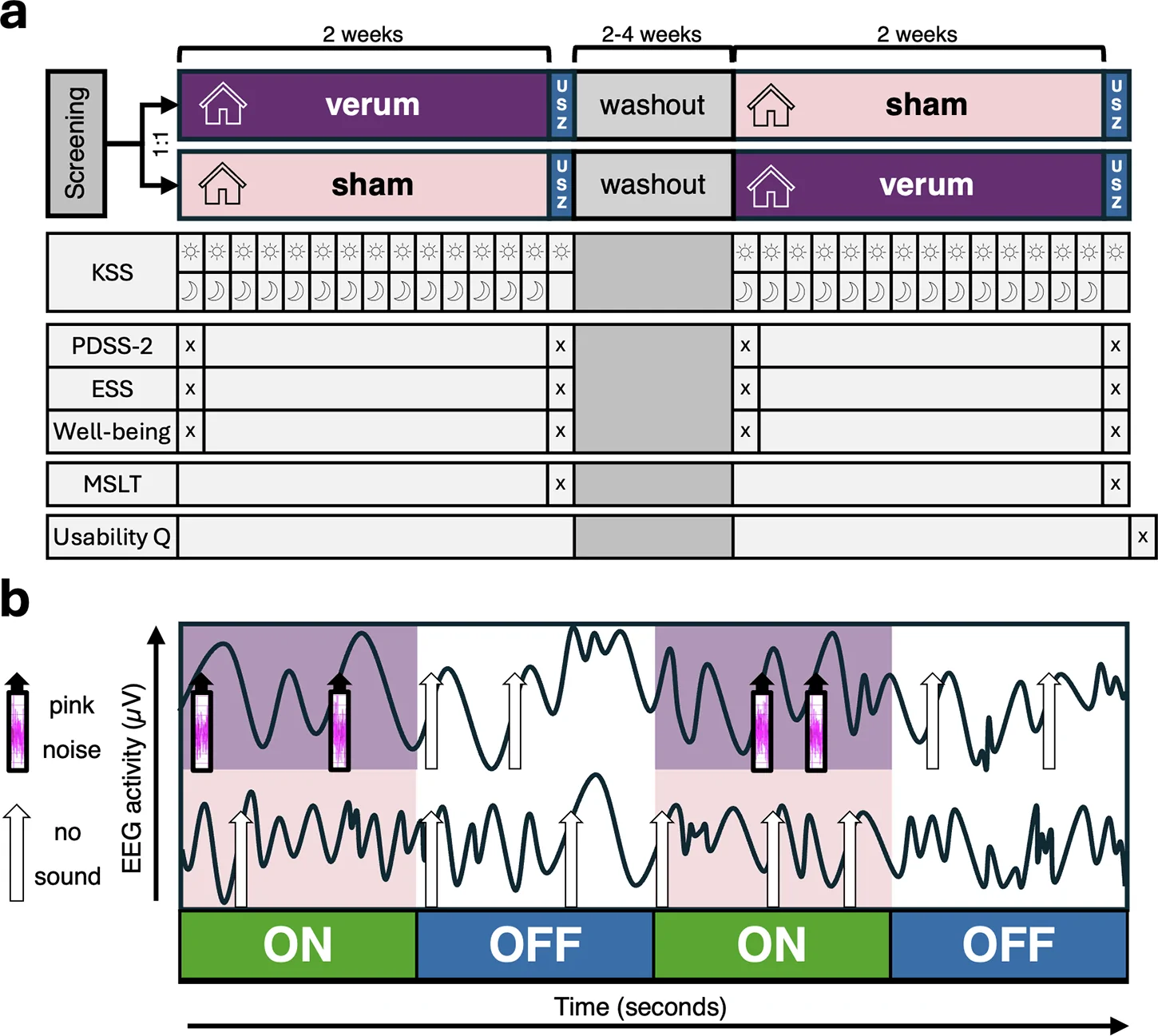
Study design and phase-targeted auditory stimulation procedure. **a** After screening, patients were randomly assigned to receive either 2 weeks of verum phase-targeted auditory stimulation (PTAS) for slow wave enhancement, followed by 2 weeks of sham stimulation or vice versa. Lower part depicts the time points of different outcome assessments, with the sun icon indicating morning, moon evening and cross throughout the day. Subjective measures included the Karolinska Sleepiness Scale (KSS), Parkinson’s Disease Sleep Scale Version 2 (PDSS-2), Epworth Sleepiness Scale (ESS), well-being (indicated on a Visual Analogue Scale). A Multiple Sleep Latency Test (MSLT) with 4 nap opportunities throughout the day was performed to assess objective daytime sleep propensity. A usability questionnaire (Usability Q) regarding self-application of PTAS was filled out at the end of study participation. Interventions and assessments prior and during interventions were performed at home, post interventions at the University Hospital Zurich (USZ). **b** PTAS procedure: Acoustic stimuli (50 ms pink noise) were presented via headphones, targeting up-phases of ongoing slow waves in alternating 6 second ON (stimulation enabled) and OFF (stimulation disabled) windows. The upper row illustrates the verum intervention, with arrows indicating time points of real (during ON windows) and potential stimulations (during OFF windows). The lower row illustrates the sham intervention, in which the same stimulation algorithm was applied as in verum, but acoustic stimuli were muted throughout.

This extension study aimed to (1) assess the feasibility, adherence, and data quality of a two-week home-based PTAS intervention, (2) reproduce initial proof-of-concept findings over a substantially longer period, and (3) evaluate the potential of PTAS to alleviate objective symptoms of daytime sleepiness. To this end, we conducted a 2 × 2-week randomised, sham-controlled, counterbalanced, double-blind cross-over study with 10 PD patients (see Fig. 1a for study design, and Table 1 for patient characteristics). A portable, self-applicable device (Tosoo Sleep Band AXO, Tosoo AG, Switzerland) was used for at-home sleep recording and PTAS administration (see Fig. 1b for PTAS administration).

**Table 1:** Patient characteristics

Characteristic	Baseline (*n* = 10)
Age (years)	60 ± 8 (52, 76)
Sex (female)	4 (40%)
Disease duration (years)	8.4 ± 6.1 (2, 20)
H&Y	2 (100%)
LED (mg/d) - levodopa-based therapy - dopamine agonists - MAO-B-inhibitors	614.8 ± 280.8 (175, 1038) - 474.8 ± 222.0 - 110.0 ± 120.3 - 30.0 ± 48.3
PDSS-2	13.2 ± 6.5 (4, 22)
PDSS-2 subscale	6.7 ± 3.9 (1, 11)
KSS (morning)	4.1 ± 1.7 (1, 6)
KSS (evening)	4.2 ± 1.6 (2, 6)
ESS	9.7 ± 4.4 (2, 14)
ESS > 10 (EDS)	5/10 patients (50%)
MDS-UPDRS III	24.7 ± 8.4 (10, 40)
MoCA	27.6 ± 1.6 (25, 30)

Continuous variables are presented as mean ± standard deviation (SD) and range, categorical variables are shown as n (%). H&Y: Hoehn and Yahr Stage. LED: Levodopa equivalent dose. MAO: Monoamine oxidase. PDSS-2: Parkinson’s Disease Sleep Scale Version 2 (range 0–60; scores ≥ 18 suggesting clinically relevant sleep disturbance). PDSS-2 subscale: Items 1-3 and 14 (higher scores = worse sleep quality). KSS: Karolinska Sleepiness Scale (range 1–9; higher scores = greater sleepiness). ESS: Epworth Sleepiness Scale (range 0–24; scores > 10 suggesting EDS (Excessive Daytime Sleepiness)). MDS-UPDRS III: Unified Parkinson’s Disease Rating Scale part III (motor examination, higher scores = more impairments). MoCA: Montreal Cognitive Assessment (range 0-30; higher scores = better cognitive function).

The intervention was well tolerated, with all adverse events being non-serious, mild in severity and fully resolved without lasting effects. The ease of use, comfort, and instructional support were evaluated positively overall, even though the range of responses was large for some items (Table 2). We successfully recorded 271 out of 280 nights (97%), of which 267 (98%) yielded data of sufficient quality for further analyses. Non-recorded nights were primarily attributable to user-related factors, including forgetting to wear the device (*n* = 2), occasional technical issues (*n* = 2) and incorrectly connected electrodes (*n* = 5). Insufficient data quality was either caused by device malfunction (*n* = 2) or a lack of sufficient sleep (*n* = 2). In addition to the high proportion of successfully recorded nights, personalisation of algorithm settings for online sleep detection by the device resulted in a satisfactory high number of PTAS stimuli being applied (average of 2297 ± 990 stimuli per night with a maximum volume of 64.4 ± 6.4 dB).

**Table 2:** Usability questionnaire responses

		Median	Mode (% of responses)	Mode (verbal category)	Range
1 Ease of use					
	I had difficulty following the procedure for using the device correctly.	0	0 (80%)	Strong disagreement	0–1
	How long did it take you at the end of the intervention to put on the sleep device?	6.5 minutes	10 minutes (40%)	-	4–10
2 Comfort and Physical Usability					
	I had difficulty falling asleep with the device.	1	0 (30%)	Strong disagreement	0–4
	I found it difficult to walk around with the device.	1	0 (40%)	Strong disagreement	0–2
	The headband disturbed my sleep.	2	2 (40%)	Rather disagreement	0–5
	The device emitted sounds that woke me up more often during the night.	0	0 (90%)	Strong disagreement	0–1
3 Overall					
	I felt very safe using the device.	5	5 (70%)	Strong agreement	0–5
	Do the manual and instruction sheet provide sufficient information to use the intervention device properly?	5	5 (60%)	Strong agreement	1–5
	Can you imagine using the intervention device outside of the study?	2.5	3 (30%)	Rather agreement	0–5

Questions were grouped into three categories, with individual questions nested under each category. Responses were rated on a Likert-type scale, where 0 = “not at all/strong disagreement” and 5 = “very/strong agreement”. For each question, the median response, the statistical mode (expressed as % of responses), the corresponding verbal category of the mode, and the observed range of responses are reported, unless otherwise indicated. No missing values occurred.

**Fig. 2:**
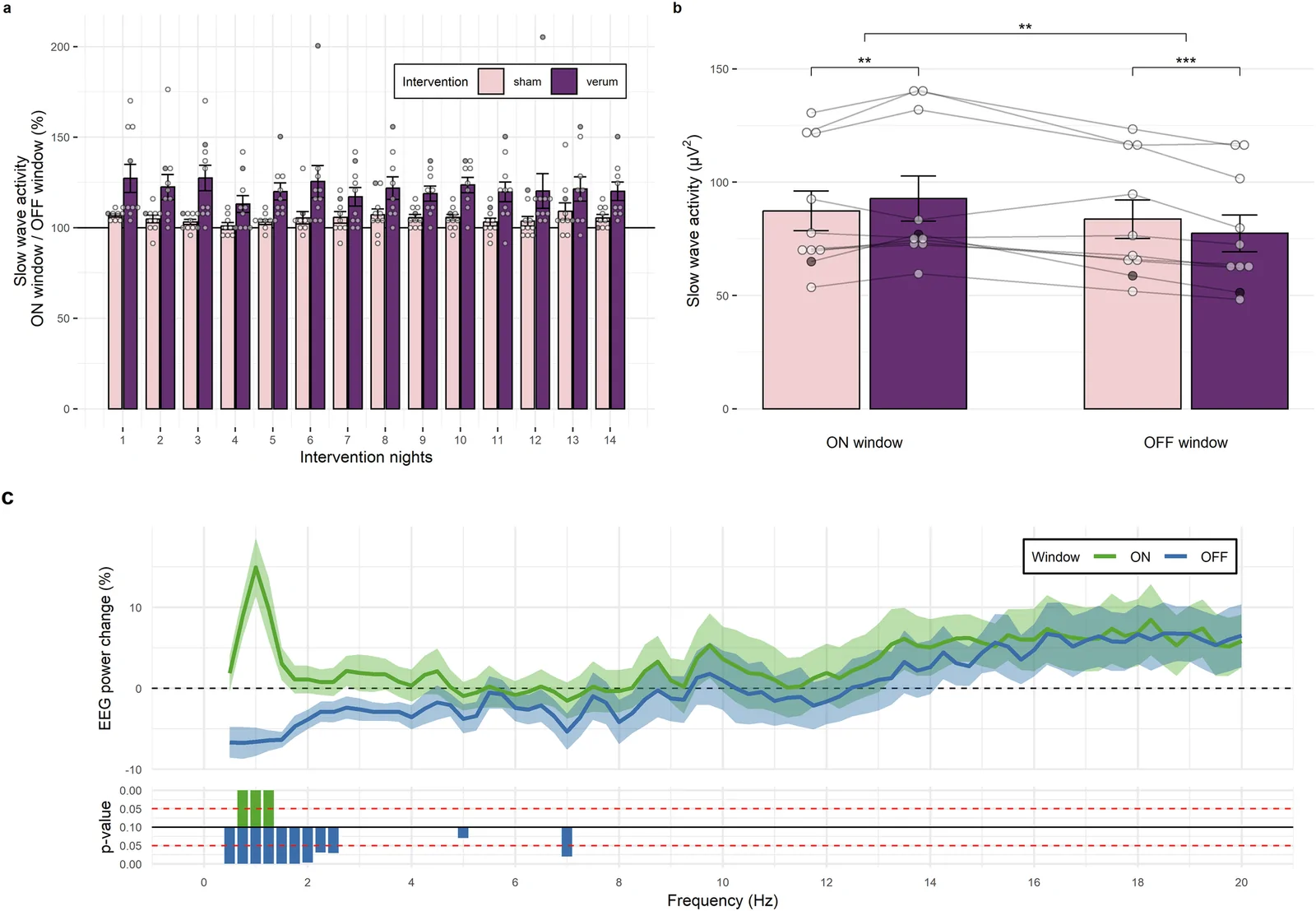
The effect of phase-targeted auditory stimulation (PTAS) on slow wave activity (SWA). **a** Bars show mean slow wave activity (SWA, 0.75–4.5 Hz) in ON windows with one or more stimulations relative to the subsequent OFF windows (100%) for each intervention night. Error bars represent the standard error of the mean (SEM), and individual data points are overlaid. The black dot indicates a participant with slightly different stimulation settings (see Methods). The linear mixed-effects model revealed a significant main effect of *intervention* (verum vs. sham, F_(1,249.33)_ = 27.81, *p* ≤ 0.001), but no *intervention x night* interaction (F_(1,249.16)_ = 0.28, *p* = 0.594), indicating that the stimulation effect on SWA was stable across nights. **b** Bars display mean slow wave activity (SWA, 0.75–4.5 Hz) for ON windows with one or more stimulations and the subsequent OFF windows, separately for verum and sham interventions. Error bars represent the standard error of the mean (SEM). Lines connect repeated measures from the same participant, and individual observations are overlaid as dot plots. The black dot indicates a participant with slightly different stimulation settings (see Methods). Stars indicate significance levels, with three representing *p* ≤ 0.001, and two representing *p* ≤ 0.01. The linear mixed-effects model indicated a significant *intervention* (verum vs. sham) *x window* (ON vs. OFF) interaction (F_(1,499.99)_ = 24.92, *p* ≤ 0.001). Post-hoc testing using estimated marginal means showed higher SWA in verum intervention in ON windows (t_(500)_ = -2.789, *p* = 0.004) and lower SWA in verum intervention in OFF windows (t_(500)_ = 4.145, *p* ≤ 0.001). **c** The *y*-axis displays the relative differences in normalised EEG power during verum across frequency bins between 0.5 Hz and 20 Hz for ON windows with one or more stimulations and the subsequent OFF windows. Values are expressed relative to the corresponding ON and OFF windows during sham. Solid lines represent group means, and shaded areas indicate the standard error of the mean (SEM). Lower panel: corresponding *p*-values obtained from linear mixed effects models fitted at each frequency bin, with Kenward-Roger and Benjamini–Hochberg correction. Horizontal red dashed lines denote thresholds for significance (*p* ≤ 0.05) and trend-level effects (0.05<*p* ≤ 0.10).

Throughout the duration of the intervention, verum auditory stimulation resulted in increased SWA (spectral power between 0.75 and 4.5 Hz) during time windows in which stimulation was enabled (ON windows) relative to windows in which stimulation was disabled (OFF windows) (Fig. 2a). The effect of PTAS on SWA differed between ON and OFF windows, with ON window SWA being higher in the verum intervention than in sham, whereas OFF window SWA was significantly lower in verum than in sham (Fig. 2b). The increase in ON windows was accentuated in low frequencies (0.75–1.25 Hz), whereas the decrease was spread across a wider frequency range (Fig. 2c). There were no signs of changes in sleep macrostructure as determined by an automatic scoring algorithm (Supplementary Table 1).

**Fig. 3:**
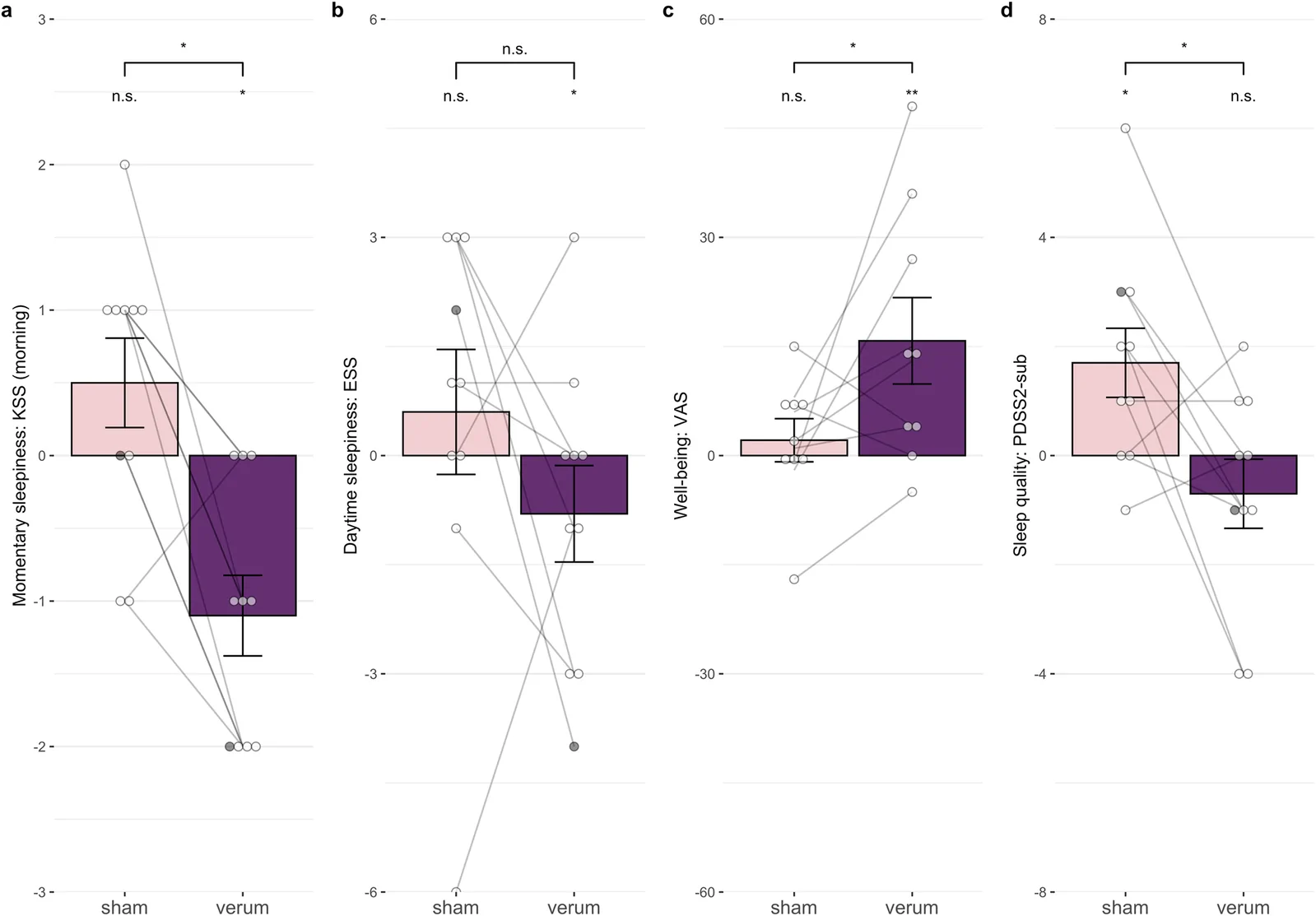
Changes in subjective outcomes from before to after intervention. Bars show group means ± the standard error of the mean (SEM); dots represent individual delta values from before to after intervention; grey lines connect repeated measures from the same participant. Data were analysed with a linear mixed-effects model and subsequent pairwise comparisons using estimated marginal means testing the simple effect of timepoint if there was a significant interaction effect. Across subplots, the black dot indicates a participant with slightly different stimulation settings (see Methods for details). Stars indicate significance levels, with two representing *p* ≤ 0.01, one representing p ≤ 0.05, and “n.s.” representing non-significant with *p* > 0.05. **a** Change in momentary sleepiness (Karolinska Sleepiness Scale, KSS, *n* = 10) was different between verum and sham (F_(1,25.58)_ = 7.82, *p* = 0.010) with verum intervention decreasing scores significantly (t_(__25.3)_ = 2.72, *p* = 0.012), whereas sham intervention did not alter scores significantly (t_(25.3)_ = -1.24, *p* = 0.228); **b** Change in daytime sleepiness, Epworth Sleepiness Scale (ESS, *n* = 10) was not significantly different between verum and sham (F_(1,23.49)_ = 1.46, *p* = 0.239); **c** Change in well-being, indicated on a Visual Analogue Scale (VAS, *n* = 9) was significantly different between verum and sham (F_(1,21.27)_ = 4.38, *p* = 0.049), with verum intervention increasing scores significantly (t_(22.2)_ = -3.417, *p* = 0.002), whereas sham intervention scores did not change (t_(22.2)_ = -0.457, *p* = 0.652). **d** Change in sleep quality (Parkinson’s Disease Sleep Scale Version 2 subscale, PDSS2-sub, *n* = 10) was significantly different between verum and sham (F_(1,25.60)_ = 6.35, *p* = 0.018), with verum intervention scores not showing a significant change (t_(25.2)_ = 1.04, *p* = 0.308), whereas sham intervention scores were increasing (t_(25.2)_ = -2.53, *p* = 0.018).

Subjectively, the change of momentary sleepiness assessed with the Karolinska Sleepiness Scale (KSS) differed significantly between interventions in the morning (Fig. 3a), but not in the evening (F_intervention_ = 0.130, *p* = 0.722). Subsequent post-hoc testing showed no change in morning sleepiness from before to after sham intervention, whereas it decreased after verum intervention. While measures of subjective daytime sleepiness over two weeks assessed with the Epworth Sleepiness Scale (ESS) did not reach statistical significance (Fig. 3b), it was directionally consistent with these findings.

For subjective well-being, the change on Visual Analogue Scale (VAS) values differed significantly between interventions (Fig. 3c), with no significant change in well-being from before to after sham intervention but increased subjective well-being after verum intervention.

For subjective sleep quality assessed with a sub-score of the Parkinson’s Disease Sleeping Scale (PDSS-2), the change differed significantly between interventions (Fig. 3d), with subjective sleep quality being negatively affected by sham intervention, while no deterioration was observed by verum intervention.

**Fig. 4:**
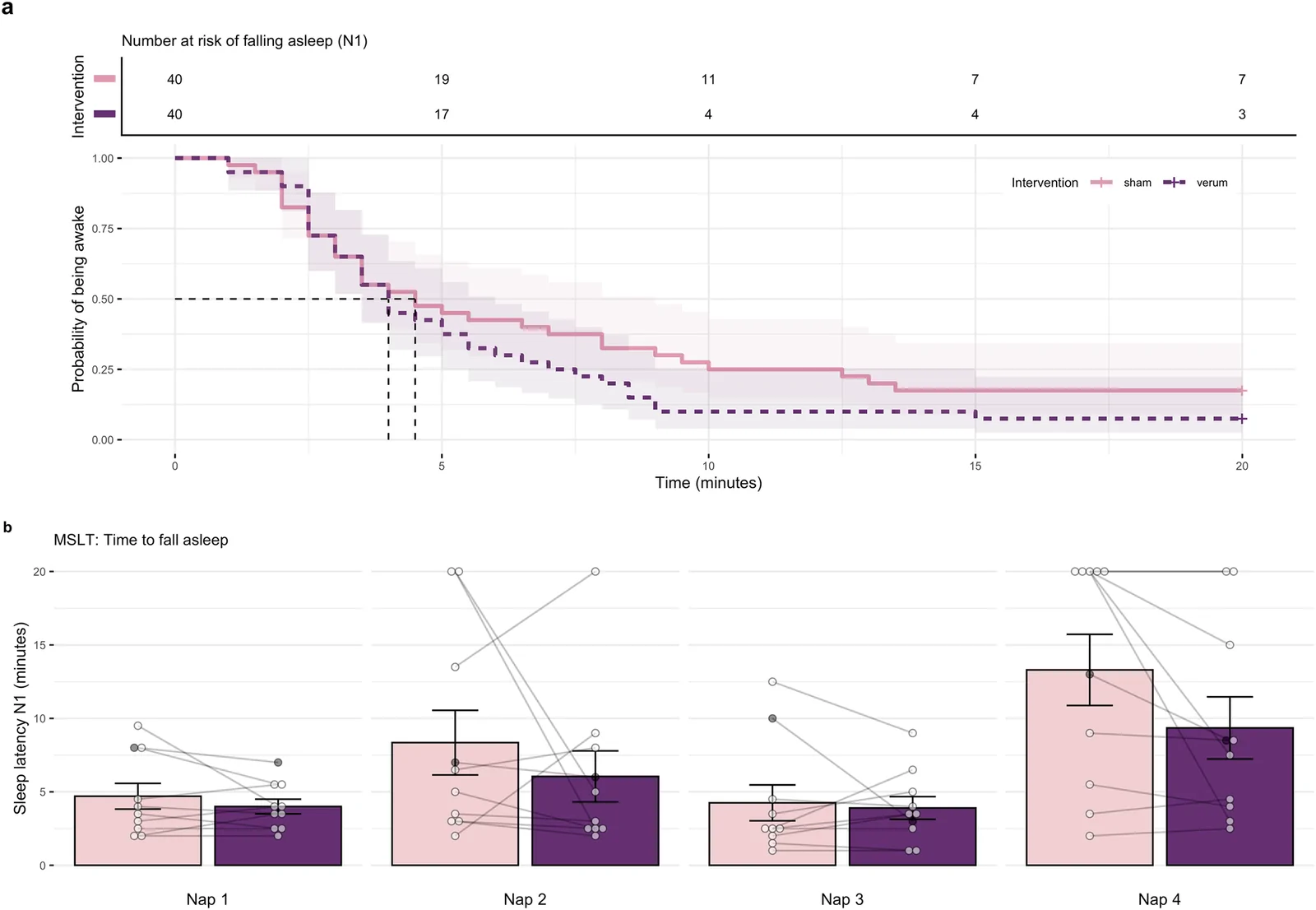
Changes in objective sleepiness after intervention. **a** Multiple Sleep Latency Test (MSLT). The Kaplan–Meier survival curve illustrates the probability of remaining awake during the MSLT after verum and sham intervention across four nap opportunities. Black dashed line: median time to fall asleep. Shaded areas indicate the 95% confidence intervals. The table on top shows the number of participants not yet fallen asleep at each time point. Cox mixed-effects survival model (*n* = 80, events=70) revealed no significant main intervention effect (verum vs. sham, HR = 0.56, 95% CI [0.05, 6.19], *p* = 0.64) or interaction for intervention × nap number (HR = 1.65, 95% CI [0.59, 4.69], *p* = 0.34). **b** Sleep latencies to N1 (sleep stage non-rapid eye movement (NREM) sleep 1) across naps in the MSLT for each of the four nap opportunities (bars = mean, dots = individual participants, error bars = the standard error of the mean (SEM)). The linear mixed-effects model showed no significant main effect of intervention (verum vs. sham, *β* = 0.13 min, 95% CI [–5.24, 5.49], *p* = 0.96) and no significant intervention × nap number interaction (*β* = –0.78 min, 95% CI [–2.74, 1.18], *p* = 0.43). The black dot indicates a participant with slightly different stimulation settings (see Methods).

Objective daytime sleep propensity assessed with the Multiple Sleep Latency Test (MSLT) did not indicate a significant intervention effect on time to fall asleep (Fig. 4).

Taken together, the present study demonstrates that self-administered, at-home PTAS in PD patients over two weeks is feasible, with excellent adherence and data quality. Application of PTAS reliably increased SWA, particularly in lower frequencies. Participants reported subjective improvements in their morning sleepiness, well-being, and unchanged sleep quality. However, no significant effects on daytime sleep propensity were observed.

The increase of SWA in low frequencies during stimulation windows, together with a subsequent broadband reduction during non-stimulation windows, without evidence for changes in sleep macrostructure is in line with other auditory deep sleep stimulation studies (e.g., refs. ^14,15,17^). The smaller SWA increase compared to the proof-of-concept study^17^ may reflect lower volume settings, calibrated to each participant’s minimal perceptual threshold in the current study. Notably, we did not see a reduction of the stimulation effect across two weeks, suggesting the absence of habituation. This is again in line with results from a study with healthy elderly^16^ and supports the potential of PTAS for sustained therapeutic use.

Unlike in some pharmacological studies^9,10,18,19^, our findings show no effect on objective (MSLT) or subjective daytime sleep propensity (ESS), as commonly used to define EDS. Reasons may be that pharmacological studies targeting EDS typically span longer periods, i.e., six to eight weeks in the above mentioned examples. Also, EDS was not an inclusion criterion. Consequently, only half of the participants exhibited clinically relevant ESS scores at baseline.

In contrast, we observed positive findings in other patient-reported outcomes (PROs), particularly on morning subjective sleepiness (KSS). As a state-sensitive measure, the KSS reveals the intensity with which sleepiness is subjectively perceived, rather than the mere tendency to fall asleep in specific situations. This effect may precede changes detectable in more stable measures of daytime sleep propensity (ESS, MSLT). Additionally, subjective sleep quality remained stable in the verum intervention but declined in the sham intervention. This suggests that active PTAS may offset potential discomfort from device use, supporting its overall tolerability.

PROs are essential for an intervention’s effectiveness and acceptability, since subjective improvements not only matter clinically but also foster treatment adherence - patients in routine practice are more likely to stick with a regimen when they experience tangible, immediate benefits^20,21^. In our study, several further factors likely contributed to the high feasibility and adherence. This included clear instructions and training, continuous support via phone with 24/7 availability, patient motivation, device usability, and daily data monitoring through remote access. Usability issues observed in this study have been addressed in later generations of the sleep device.

Limitations of this study include the selective inclusion of patients that would show a response to PTAS during the screening procedure. This strategy helps to reveal its potential but prevents conclusions about efficacy. The exclusion of patients with more severe disease (Hoehn and Yahr stage > III), deep brain stimulation implants and the small sample size limit generalisability, with the latter also restricting statistical power. Despite the longer intervention period compared to the proof-of-concept study^17^, the two-week duration remains relatively short. These factors may have contributed to the lack of observable effects on objective daytime sleepiness. Longer studies with extended intervention periods and targeted inclusion of patients with marked EDS are needed to evaluate clinical efficacy and durability of PTAS effects.

Growing evidence supports a bidirectional relationship between neurodegeneration and sleep: while the disease often produces sleep-wake disturbances^22^, SWS is associated with a slower progression of motor symptoms in PD^23^, and preclinical data suggests a role in disease pathophysiology^24^. This makes deepening sleep a promising strategy to interfere with disease progression. Viewed in this light, the results of our study position the home-based PTAS application as a suitable candidate to test the impact of deep sleep modulation on disease progression in long-term studies.

Altogether, this study demonstrates the feasibility and strong patient adherence to home-based, self-applied PTAS in PD. The portable sleep stimulation device proved technically reliable for both recording and stimulation without habituation. Despite limited objective effects, participants reported subjective improvements, alongside persistent modulation of SWA. These findings support the feasibility of PTAS as a non-pharmacological intervention and lay the groundwork for longer-term studies and real-life clinical applications aimed at reducing subjective sleepiness in patients with PD. Prolonged intervention periods may be necessary to determine effects on objective daytime sleepiness and may even be applied in the future to assess its disease modifying potential in PD.

## Methods

### Ethics

The study was conducted according to the Declaration of Helsinki principles and approved by the Cantonal Ethics Committee of Zurich (reference: BASEC 2020-02298) and Swissmedic, the Swiss agency for drugs and therapeutic products (reference 10000791), and initially registered on 29.01.2021 at ClinicalTrials.gov (NCT04736017, https://clinicaltrials.gov/study/NCT04736017?id=NCT04736017) and KOFAM (SNCTP000004218,https://www.humanforschung-schweiz.ch/de/studiensuche/studien-detail/54276#query=SNCTP000004218). Study protocols and statistical analysis plans can be found on ClinicalTrials.gov. Participants provided written informed consent prior to study inclusion and were reimbursed for their inconvenience and time taken in the study participation with CHF 1000.- upon completion of the study. Harms were defined and assessed according to standard clinical trial procedures, including adverse events (AEs) and adverse device effects (ADEs).

### Study design

This extension study to Schreiner et al.^17^ was a single-centre, randomised, sham-controlled, counter-balanced, double-blind, cross-over trial with a 1:1 allocation ratio conducted at the Department of Neurology, University Hospital Zurich, Switzerland. Data collection took place between April 2021 and July 2022 and included six participants from the aforementioned proof-of-concept study^17^ and four newly enrolled participants. There was no direct patient or public involvement in the design, conduct, or reporting of this trial.

After completion of the first patient, some adaptations to the study protocol were made with regards to outcomes assessed and specifics in the stimulation protocol (see description of stimulation protocol below). We still included the patient in the analysis where possible but marked the corresponding data point in the figures.

While the proof-of-concept study limited the intervention period to three days, the current study design extended the intervention period to 14 consecutive days, separated by a wash-out period of at least two weeks (range 15 to 29 days). Participants applied self-administered, at-home PTAS, supported by tablet-based or computer-based questionnaires. Participant compliance was monitored daily by checking for successful data transmission and through an online platform that tracked questionnaire completion. Additionally, two on-site visits were performed after both interventions for the objective outcome assessment. Participants in both interventions continued to receive their standard PD treatment throughout the trial. No additional experimental co-interventions were provided.

### Participants

Participants were recruited through multiple channels, including printed advertisements in the magazine of the Parkinson Schweiz organisation, local patient support groups, the movement disorders outpatient clinic at the Department of Neurology, University Hospital Zurich, as well as selected neurological practices or physiotherapists across Switzerland. Initial screening was conducted during a visit in the outpatient clinic at the Department of Neurology, University Hospital Zurich and in the participants’ homes, where experimenters introduced the study protocol, delivered the sleep device and a tablet, and provided comprehensive instructions along with hands-on training to ensure participants were comfortable using the equipment. Ongoing support was provided throughout the study via regular phone calls. Additionally, participants had 24/7 access to assistance and could contact the study team at any time if issues arose.

Screening nights were conducted in participants’ homes to configure individual parameters of the sleep detection and PTAS algorithm and to ensure suitability for at-home application. Data collected during these nights were used to assess the presence of a stimulus-induced response in the EEG upon PTAS and to titrate stimulation volume if necessary. Participants who showed a persistent lack of response (despite more than 500 stimuli being delivered and increasing volume)—defined as the absence of a visible event-related potential (ERP) averaged across all available stimuli in response to auditory stimulation—were excluded from further participation. Additional inclusion and exclusion criteria were comparable to the proof-of-concept study^17^, with the inclusion criteria encompassing a diagnosis of moderate PD (Hoehn & Yahr stages II-III), stable Parkinson medication, plus the ability to understand and follow the study procedures. In contrast to Schreiner et al.^17^, self-reported sleep disturbances were no inclusion criterion. The main exclusion criteria were the presence of other neurologic, psychiatric, or sleep disorders (such as sleep apnea or restless leg syndrome, Parkinsonism without response to levodopa, and cognitive impairment (defined as a Montreal Cognitive Assessment (MoCA) score below 24). Sleep apnea was screened at home using the Alice NightOne diagnostic system.

**Fig. 5:**
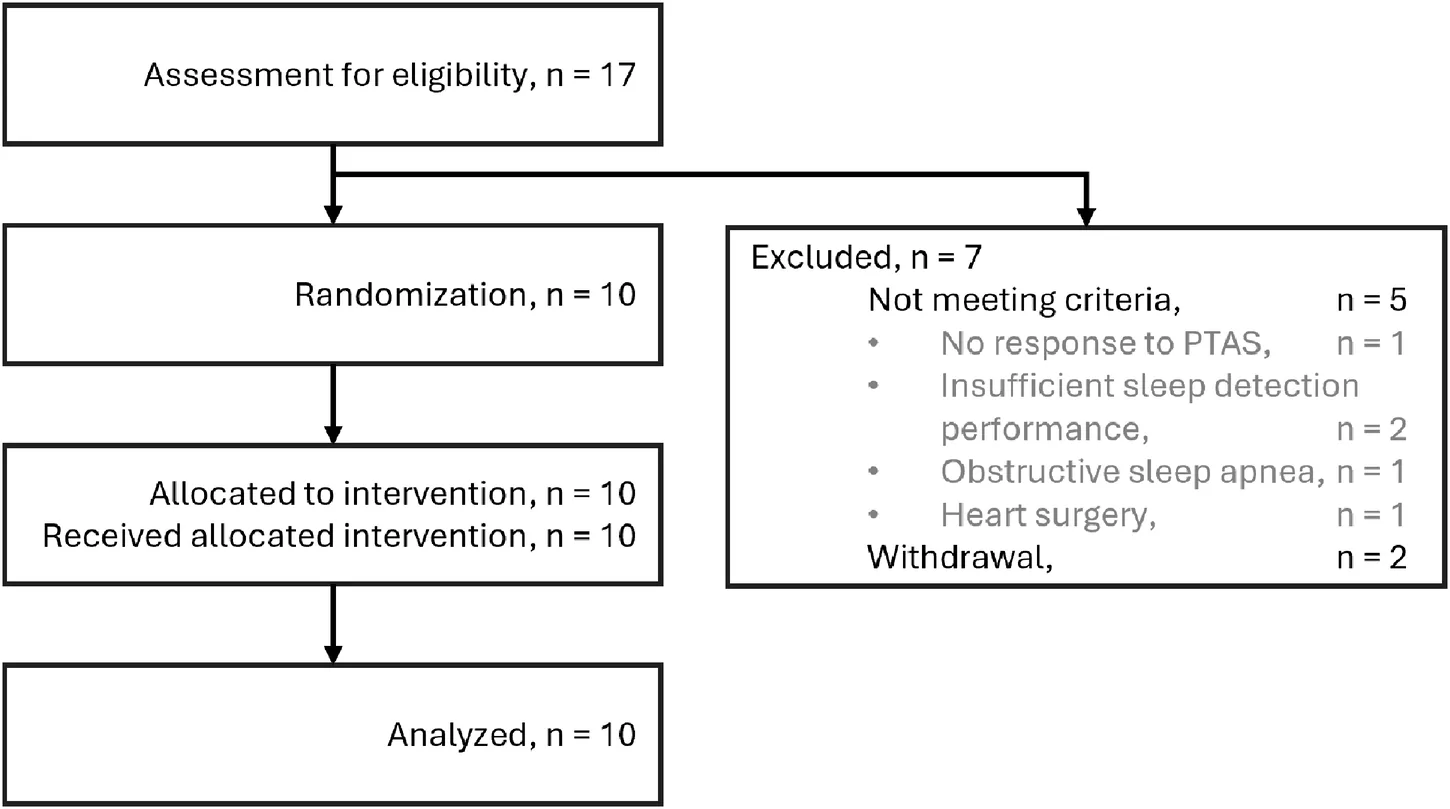
Patient flow chart. Of 17 participants undergoing screening, 10 were randomised, allocated to intervention, received respective interventions, and were subsequently analysed. Of the 7 excluded participants during screening, 2 withdrew themselves from the study and 5 did not meet the criteria, most often (*n* = 2) due to an insufficient sleep detection performance despite personalisation of sleep detection thresholds. PTAS: Phase-targeted auditory stimulation. See methods section for more details.

The participant recruitment flowchart shows the number of participants from inclusion until analysis (Fig. 5). Ten patients were successfully screened and completed both intervention periods.

### Randomisation & blinding

An experimenter without direct participant contact and not involved in data acquisition, processing, or analysis performed the randomisation. Simple, unrestricted randomisation was used without any stratification – only counterbalancing was ensured. Allocation was implemented in a decentralised manner: the randomiser programmed a TSB Axo stimulation device according to the assigned code, setting it to either verum (active stimulation) or sham (no stimulation). The device settings and randomisation table were not accessible to other study staff (i.e., experimenters in contact with participants) throughout study conductance. Also, participants were blinded to the intervention condition. Experimenters followed the same procedures in both intervention and identical devices with the same instructions and support were used across both intervention phases. Unblinding was performed for the statistical data analysis after data pre-processing (e.g., correction of automatic sleep staging).

### Phase-targeted auditory stimulation (PTAS)

Similar to the proof-of-concept study^17^, acoustic stimuli (50 ms pink noise) were presented via headphones, targeting up-phases of slow waves in an ON-OFF windowed fashion (meaning alternating 6-second windows with stimulation enabled/disabled throughout detected NREM sleep, see Fig. 1b). The wearable system used for sleep recording and modulation was the TSB AXO (Tosoo Sleep Band AXO, Tosoo AG, Switzerland), a further development of the previously used Mobile Health Systems Lab (MHSL) SleepBand (MHSL-SB)^16,17,25^. The system used a sampling rate of 250 Hz and recorded one single EEG signal (FpZ-A2) for real-time processing and one EOG signal for post-processing purposes.

In the study’s initial protocol (subjected to *n* = 1), we used a phase-vocoder algorithm for EEG phase estimation^26^ and applied whole-night PTAS, as in the first subgroup of participants assessed in the proof-of-concept study^17^. Due to emerging evidence at that time that this paradigm might negatively affect mood through its impact on REM sleep^16^, we subsequently adapted the protocol (subjected to n = 9) to part-night PTAS (duration individualised to each patient), being limited to the first part of the night with its predominance of deep sleep. Additionally, the EEG phase estimation algorithm of slow waves was changed to a phase-locked loop to align with the proof-of-concept study^17^, except for the minimum inter-stimulus-interval which was now set to 1 second. Additionally, we changed the personalised volume setting procedure to start at quieter volumes just above hearing threshold to counteract potential arousals^14,15^. In both protocols, the stimulation volume increased within predefined limits during deep sleep in the absence of arousals and paused stimulation if an arousal was detected. EEG analyses of the data with the initial protocol were limited to the first three hours after the delivery of the first stimulus, to align with the part-night PTAS data of the adapted protocol. While the verum intervention delivered audible (adjusted to individual hearing threshold) stimuli, the sham intervention consisted of muted stimuli.

### Sleep macrostructure

Sleep macrostructure of intervention nights was assessed using a proprietary, automated sleep-staging algorithm based on single-channel EEG (FpZ-A2), specifically tailored for the PD population. Developed using PD patient data recorded with previous versions of the same EEG system, engineered features, and expert-labelled scores, the algorithm employed a recurrent neural network for sleep stage classification. The algorithm provided staging outputs in three consolidated categories: WAKE/N1 (combining Wake and N1), NREM2/3 (combining N2 and N3), and REM sleep. The dataset comprised 142 nights from 49 PD patients: 39 patients (116 nights) were used for training and 10 patients (26 nights) for testing. On the test set, the algorithm achieved a Cohen’s kappa of 0.67 and an overall accuracy of 88%.

Finally, spectrograms of each night were compared to their scoring and manually corrected if notable irregularities were apparent in the beginning or end of the night. This was done to ensure proper identification of the sleep period. Sleep period was defined as the duration from the first NREM2/3 sleep epoch to the first epoch of final WAKE/N1, whereas WASO is defined as the time of WAKE/N1 during the sleep period. Awakenings are defined as the number of transitions from either NREM2/3 or REM epochs to WAKE/N1 during the sleep period. Sleep efficiency was calculated as the sum of NREM2/3 and REM epochs during the sleep period. REM sleep latency is reported from the start of the sleep period until the first epoch of REM sleep.

Sleep latency of daytime naps in the MSLT was assessed using standard clinical EEG montage, with scoring performed by certified sleep experts in accordance with the criteria of the AASM Manual for the Scoring of Sleep and Associated Events.

### EEG spectral analysis

Semi-automated artifact rejection was applied to NREM sleep epochs using a single-channel implementation of the High-Density-SleepCleaner artefact removal routine^27^. EEG analysis was conducted in line with Lustenberger et al.^16^ and Schreiner et al.^17^. EEG data were bandpass filtered between 0.5 and 40 Hz using a two-step finite impulse response (FIR) filter implemented via the eegfilt function from the EEGLAB toolbox^28^ in Matlab (The MathWorks, Inc., Natick, MA). Subsequently, power spectral density (PSD) estimates were computed using Welch’s method, implemented via the MATLAB’s pwelch function. A Hanning window of 4 seconds was applied with 50% overlap, resulting in a mean frequency response for each 6-second window. For latter analysis steps, only consecutive ON-OFF windows with at least one stimulation in the ON window classified as artefact-free NREM epochs were used. For consistency with previous publications mentioned above^16,17^, EEG power spectra were normalised to the average power between 0.5–20 Hz in each assessed frequency bin for the corresponding analysis (Fig. 2c).

### Subjective & objective sleep-related outcomes

Subjective momentary sleepiness was assessed twice daily (morning and evening) at home using tablet-based questionnaires featuring the Karolinska Sleepiness Scale (KSS)^29^. The morning measures of the fourteenth morning were analysed as the final outcome, given that the fifteenth morning measurement was tainted by slightly earlier wake-up times due to the participants having to travel to the study site. In one case the first KSS measurement (sham) was missing and was replaced by the first measurement of the other intervention (verum).

Subjective daytime sleepiness across 2 weeks was assessed using the Epworth Sleepiness Scale (ESS)^30^, while subjective well-being was evaluated with a Visual Analogue Scale (VAS) based on the question: ‘Overall, how satisfied have you been during the past two weeks?’. Additionally, sleep quality was evaluated through selected items from the Parkinson’s Disease Sleep Scale Version 2 (PDSS-2 subscale)^31^. The PDSS-2 is a validated self-administered questionnaire comprising three domains: nocturnal motor symptoms, PD-specific symptoms during the night, and disturbed sleep. For the present analysis, we focused on the sum of items 1–3 and 14—assessing poor sleep quality, difficulty falling asleep, difficulty maintaining sleep, and feeling tired or sleepy upon awakening—to focus on the most common PD-related sleep disturbances^32,33^. The ESS, VAS and PDSS-2 were assessed at the patient’s home pre-intervention and at the sleep laboratory of the University Hospital Zurich post-intervention. The MSLT, a clinical measure of objective daytime sleepiness, was administered to all patients at four standardised times (10:00, 12:00, 14:00, and 16:00) after the last intervention night. The sleep latency to N1 in minutes was taken as the outcome variable and was scored by certified sleep medicine specialists.

### Statistical analysis

For statistical analyses of SWA and subjective outcomes, linear mixed-effects models were applied (lmer function, *lme4* package in R), including participant ID as a random intercept to account for repeated measures within individuals.

For SWA across intervention nights (Fig. 2a; *n* = 267 nights from 10 participants), fixed effects comprised sequence (sham–verum vs. verum–sham), period (1 or 2), self-reported nocturnal sleep duration, intervention (verum vs. sham), night (1–14), and their interaction. SWA values were analysed as log10-transformed ON/OFF ratios to improve residual normality. For clarity, data are visualised as ON-to-OFF percentages.

For SWA during ON versus OFF windows (Fig. 2b; *n* = 534 windows, 267 nights × 2), the model additionally included a window × intervention interaction. EEG power spectra were log10-transformed prior to analysis. Model assumptions regarding residual distribution and homoscedasticity were verified visually (*check_model*, *performance* package). Post-hoc pairwise comparisons were conducted using estimated marginal means (*emmeans* package) without adjustment for multiple comparisons, given the limited number of planned contrasts.

For spectral analyses across frequency bins (Fig. 2c; *n* = 1580 power values from 10 participants, 2 windows, 79 frequency bins), normalised EEG power spectra were compared between interventions (verum vs. sham) separately for ON windows (containing ≥1 stimulation) and their subsequent OFF windows. Relative differences were expressed as percentage change from sham. Linear mixed effects models with Kenward-Roger (degrees of freedom) and Benjamini-Hochberg correction (false discovery rate) were fitted per frequency bin. *p* ≤ 0.05 was considered statistically significant; values between 0.05 and 0.10 were interpreted as trends.

Subjective outcomes (KSS, ESS, PDSS-2 subscales, and well-being; *n* = 36–40 observations from 9–10 participants × 2 interventions × 2 time points) were analysed using separate mixed-effects models including sequence, period, sleep duration, intervention, time point (pre/post), and their interaction as fixed effects.

To evaluate factors influencing wake probability during the MSLT, a Cox proportional hazards mixed-effects model was used (*coxme* package in R; *n* = 80 observations, events = 70) with participant ID as a random intercept. Fixed effects included self-reported nocturnal sleep duration, daytime sleepiness (ESS), intervention, nap number (1–4), and all interactions between ESS, intervention, and nap number. A survival approach was chosen because sleep onset could be right-censored if participants remained awake throughout the nap. Mean sleep latency per nap was analysed using a linear mixed-effects model with fixed effects for intervention, nap number, and their interaction, and a random intercept for participant ID. 95% confidence intervals were reported alongside point estimates. The main effect of intervention (verum vs. sham) constituted the primary comparison of interest.

Across all analyses, significance was set at α = 0.05 (two-tailed). Sample size was based on power analyses from previous studies^9,10^. No interim analyses were performed.

## Supplementary information

Supplementary information

## Data Availability

The raw datasets generated and analysed during the current study are not publicly available to protect participants’ rights according to Swiss human research law. Deidentified datasets are available from the corresponding author on reasonable request, which includes the following steps: (1) submit a methodological sound proposal describing the intended analysis and as reviewed by the authors of this publication, (2) provide proof of relevant ethical approval for the intended analysis, and (3) fulfil data protection measures according to Swiss legal requirements.

## Code availability

The underlying code for this study is not publicly available but may be made available to qualified researchers on reasonable request from the corresponding author.
